# Response Regulator Heterodimer Formation Controls a Key Stage in S*treptomyces* Development

**DOI:** 10.1371/journal.pgen.1004554

**Published:** 2014-08-07

**Authors:** Mahmoud M. Al-Bassam, Maureen J. Bibb, Matthew J. Bush, Govind Chandra, Mark J. Buttner

**Affiliations:** Department of Molecular Microbiology, John Innes Centre, Norwich Research Park, Colney Lane, Norwich, United Kingdom; Indiana University, United States of America

## Abstract

The orphan, atypical response regulators BldM and WhiI each play critical roles in *Streptomyces* differentiation. BldM is required for the formation of aerial hyphae, and WhiI is required for the differentiation of these reproductive structures into mature spores. To gain insight into BldM function, we defined the genome-wide BldM regulon using ChIP-Seq and transcriptional profiling. BldM target genes clustered into two groups based on their *whi* gene dependency. Expression of Group I genes depended on *bldM* but was independent of all the *whi* genes, and biochemical experiments showed that Group I promoters were controlled by a BldM homodimer. In contrast, Group II genes were expressed later than Group I genes and their expression depended not only on *bldM* but also on *whiI* and *whiG* (encoding the sigma factor that activates *whiI*). Additional ChIP-Seq analysis showed that BldM Group II genes were also direct targets of WhiI and that *in vivo* binding of WhiI to these promoters depended on BldM and *vice versa*. We go on to demonstrate that BldM and WhiI form a functional heterodimer that controls Group II promoters, serving to integrate signals from two distinct developmental pathways. The BldM-WhiI system thus exemplifies the potential of response regulator heterodimer formation as a mechanism to expand the signaling capabilities of bacterial cells.

## Author Summary

Two-component signal transduction systems are a primary means of regulating gene expression in bacteria. Recognizing the diversity of mechanisms associated with these systems is therefore critical to understanding the full signaling potential of bacterial cells. We have analyzed the behavior of two orphan, atypical response regulators that play key roles in controlling morphological differentiation in the filamentous bacteria *Streptomyces*-BldM and WhiI. We demonstrate that BldM activates its Group I target promoters as a homodimer, but that it subsequently activates its Group II target promoters by forming a functional heterodimer with WhiI. BldM-WhiI heterodimer formation thus represents an unusual mechanism for the coactivation of target genes and the integration of regulatory signals at promoters, enhancing the known repertoire of signaling capabilities associated with two-component systems.

## Introduction

Two-component signal transduction systems are of central importance in regulating gene expression in bacteria. Canonically they consist of a response regulator, which functions as a homodimer, and a cognate sensor histidine kinase (which may also function as a cognate phosphatase). The activity of the kinase/phosphatase is modulated in response to a perceived stimulus. The sensor kinase autophosphorylates on a conserved histidine residue, and the phosphoryl group is then transferred to a conserved aspartate in the response regulator. The addition of the phosphoryl group stabilizes a conformation of the response regulator that drives an output response, most often the activation of gene expression. However, the intrinsic modularity of these systems has allowed bacteria to evolve variations on this basic theme, including more complex multicomponent phosphorelays, and changes in the nature of the response regulator effector domain such that the output can be, for example, an enzymatic activity rather than DNA binding [1]–[3]. Recognizing the diversity of mechanisms associated with these systems is therefore critical to understanding the full potential of the signaling capabilities of bacterial cells. This study concerns the behavior of two response regulators required for morphological development in the filamentous bacteria *Streptomyces*.

When streptomycete spores germinate, one or two germ tubes emerge and grow by tip extension and branching to form an extensive, multicellular vegetative mycelium [4]–[6]. Streptomycetes differentiate by forming specialized reproductive structures called aerial hyphae, which emerge from the colony surface into the air. The formation of aerial hyphae requires the activity of a class of developmental master regulators encoded by the *bld* (bald) genes [4]–[6]. Subsequently, in the most dramatic event of the lifecycle, each multigenomic aerial hypha arrests tip growth and undergoes a massive, synchronous septation event, giving rise to ∼50–100 unigenomic prespore compartments that ultimately develop into mature, pigmented exospores [4]–[6]. The differentiation of aerial hyphae into mature spores is coordinated by the activity of a second class of developmental master regulators encoded by the *whi* (white) genes. The focus of this work is the interaction between two of these global regulators, BldM and WhiI.

BldM and WhiI are both atypical response regulators (ARRs). In canonical response regulators, the aspartate residue that is subject to phosphorylation sits in a highly conserved pocket within the N-terminal receiver domain. ARRs usually lack essential residues within this phosphorylation pocket, suggesting that their activity is not controlled by phosphorylation [7]–[11]. WhiI has a degenerate phosphorylation pocket, lacking a universally conserved lysine and one of a pair of adjacent aspartate residues essential for binding Mg^2+^ [12], [13]. Although BldM does have a conserved phosphorylation pocket, a *bldM* allele carrying a D54A substitution at the putative site of phosporylation fully complements a *bldM* null mutant [14]. Moreover, BldM could not be phosphorylated *in vitro* [14]. Finally, BldM and WhiI are both ‘orphan’ response regulators – their genes are not adjacent to a sensor kinase gene, as is most often the case for canonical response regulators. Taken together, these observations strongly suggest that BldM and WhiI are not controlled by phosphorylation as part of conventional two-component systems. BldM and WhiI both belong to the NarL/FixJ subfamily of response regulators. *bldM* and *whiI* are found in all sequenced streptomycete genomes and their chromosomal context is conserved throughout. Strikingly, the amino acid sequence of BldM is 100% identical across all sequenced streptomycetes (the only response regulator that is 100% identical across all streptomycetes). WhiI is at least 93% identical, with amino acid variations found mainly in the linker region between the degenerate receiver domain and the DNA-binding domain. All sequenced WhiIs have a degenerate phosphorylation pocket.

During differentiation, the *whiI* and *bldM* genes are activated by two cognate, development-specific sigma factors, σ^WhiG^ and σ^BldN^, respectively. *whiI* expression is activated from a single σ^WhiG^ target promoter, and thus *whiI* is not expressed in a *whiG* mutant [12]. σ^BldN^ directs transcription of the *p1* promoter of *bldM* (the other promoter, *bldMp2*, is σ^BldN^-independent, and so transcription of *bldM* is developmentally activated from only one of its two promoters in a *bldN* mutant [15], [16]). In addition to *bldM*, the other key targets of σ^BldN^ are the genes encoding the chaplins and rodlins, the major proteins of the hydrophobic sheath that coats the aerial hyphae and spores in *Streptomyces* [16]–[19].

*Streptomyces venezuelae* has recently emerged as an attractive new model system for the analysis of *Streptomyces* development because it sporulates in liquid culture [16], [20]. Here we take advantage of the *S. venezuelae* system to apply global microarray transcriptional profiling and ChIP-Seq to characterize the BldM and WhiI regulons. Through this route we go on to show that a key stage in *Streptomyces* development is controlled by response regulator heterodimer formation between BldM and WhiI, and to greatly expand our understanding of the regulatory network that controls morphological differentiation in these multicellular bacteria. The BldM-WhiI system thus exemplifies the potential of response regulator heterodimer formation as a mechanism to expand the signaling capabilities of bacterial cells.

## Results

### BldM directly activates the expression of key developmental genes

Having established conditions in which *S. venezuelae* sporulates abundantly in liquid culture [16], [20], immunoblotting of samples taken at 14, 15 and 16 h of growth in MYM liquid sporulation medium showed that BldM was abundant at each of these time points (**Figure S1**). To gain greater insight into BldM function, we defined the genome-wide BldM regulon using ChIP-Seq. As described in *Materials and Methods*, wild-type *S. venezuelae* was subjected to formaldehyde cross-linking, lysis and sonication after 16 h of growth. After immunoprecipitation using a BldM-specific polyclonal antibody, the resulting DNA was subjected to deep sequencing. As a negative control, a ChIP-Seq experiment was performed on the congenic *bldM* null mutant. Several well-characterized developmental loci, including *ssgR*, *rshA*, *smeA*-*sffA*, *whiB* and *whiE*, were among the direct BldM targets identified.

Next, in order to determine how BldM influences the expression of its target genes, wild-type *S. venezuelae* and the congenic *ΔbldM* mutant were subjected to time-resolved, genome-wide transcriptional profiling during vegetative growth and sporulation. Strains were grown under the same conditions used for the ChIP-Seq experiments. RNA samples were prepared at 2-hour intervals from 8 to 20 hours, by which time sporulation was nearing completion, and following cDNA synthesis and labeling, samples were hybridized to Affymetrix DNA microarrays. Three independent biological replicates were performed for each strain, and analysis of the resulting data showed that the expression of 131 direct BldM targets was significantly down-regulated in the *ΔbldM* mutant (p<0.01) in comparison to the wild type. In contrast, only six genes were up-regulated in the *ΔbldM* mutant (p<0.01) in comparison to the wild type. These results suggest that BldM functions mainly as a transcriptional activator.

### Many BldM target genes cluster into two discrete groups based on *whi* gene dependency

**Figure 1:**
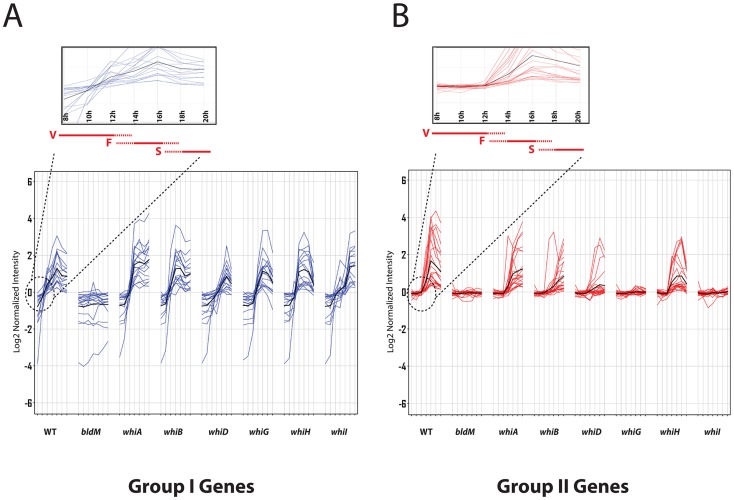
Microarray transcription profiles of (A) Group-I and (B) Group-II genes in wild-type *S. venezuelae* during submerged sporulation and in congenic *ΔbldM*, *ΔwhiA*, *ΔwhiB*, *ΔwhiD*, *ΔwhiG*, *ΔwhiH* and *ΔwhiI* null mutants grown under identical conditions. RNA samples were prepared at 2-hour intervals from 8 to 20 hours, by which time sporulation was nearing completion. For each panel, the x-axis indicates the age of the culture in hours, and the y-axis indicates the per gene normalized transcript abundance (log_2_). Group-I genes (blue) depend on *bldM* but are independent of all the *whi* genes. Group-II genes (red) depend on *bldM* but also depend *whiG* and *whiI*. The average expression profile is indicated by the black line. Group I genes are activated at least two hours earlier than Group-II genes (see insets). Red bars indicate vegetative growth (V), fragmentation of mycelium (F), and spore formation (S).

We next determined the time-resolved transcriptional profiles of the BldM target genes in seven constructed white mutants: *ΔwhiA*, *ΔwhiB*, *ΔwhiD*, *ΔwhiG*, *ΔwhiH* and *ΔwhiI*. Strikingly, many of the BldM target genes clustered into two well-defined groups according to their dependencies on the *whi* genes. Group I genes consisted of developmentally induced genes that depend on *bldM*, but were activated normally in all the *whi* mutants (**Figure 1A** and **Table S1**). Group II BldM target genes were also developmentally induced, but depended not only on *bldM*, but also on *whiG* and *whiI* (**Figure 1B** and **Table S2**).

### Group I genes-identification of a consensus Group I BldM binding site

**Figure 2:**
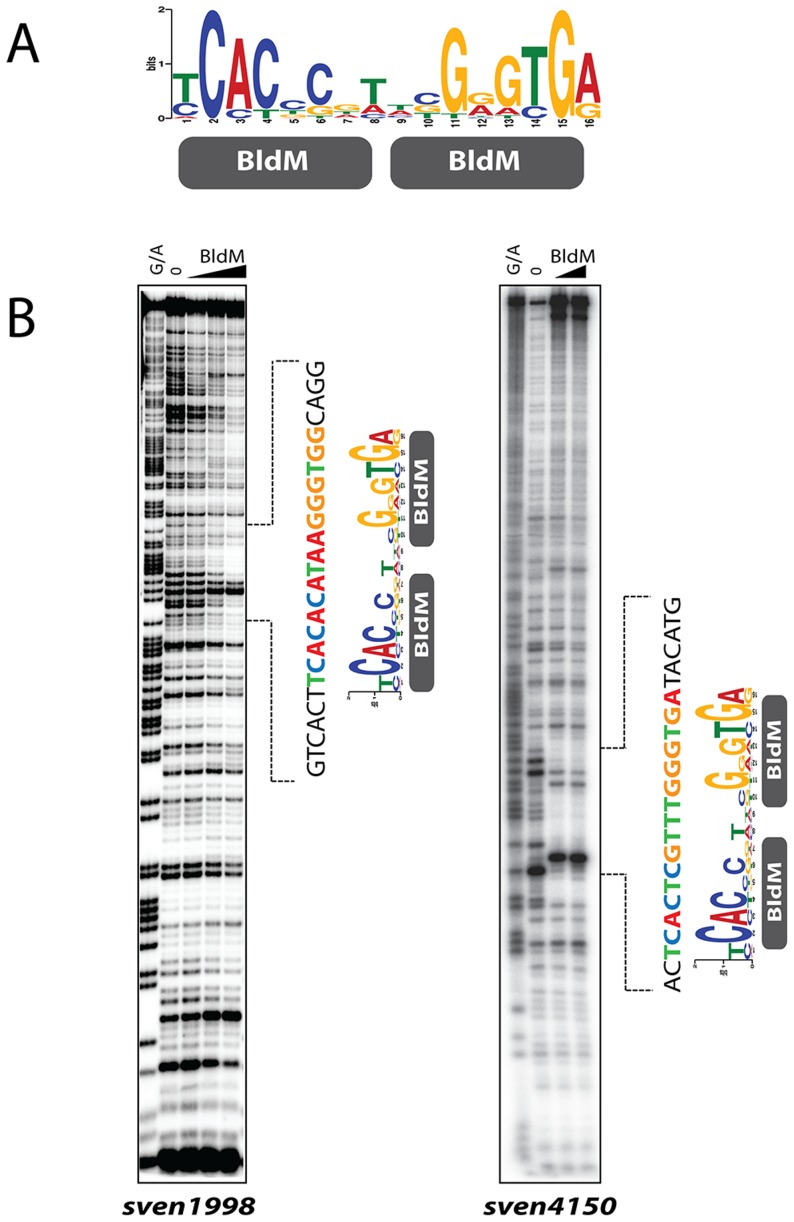
BldM binds to the *MEME*-predicted Group-I consensus sequence. A. The *MEME*-predicted palindromic Group-I consensus sequence. The height of the letters in the sequence logo, in bits, is proportional to the frequency of the A, C, T, or G nucleotides at each position of the motif. B. DNase I footprinting analysis of BldM on two Group-I promoters: *sven1998* and *sven4150*. The BldM protein concentrations used were 0, 100, 250 and 500 nM for *sven1998* and 0, 250 and 500 nM for *sven4150*. G+A indicates the Maxam and Gilbert sequence ladder. The protected regions are indicated by dotted lines and the positions of the *MEME*-predicted Group I binding motif are shown.

To gain further insight into Group I binding sites, we fed the sequences of Group I promoter regions into the MEME algorithm [21] to search for over-represented sequences, using as input the entire intergenic region in each case. This analysis revealed a well-conserved copy of a 16 bp palindromic sequence, 5′-TCACcCgnncGgGTGA-3′, for which the sequence logo is shown in **Figure 2A**. The palindromic nature of this sequence would be consistent with BldM binding as a homodimer to Group I promoters.

To test the validity of the MEME output, and to confirm and extend the ChIP-Seq analysis, we overexpressed and purified BldM from *E. coli* as a soluble, N-terminally His_6_-tagged protein. The resulting BldM protein was used in DNase I footprinting analysis on the intergenic regions upstream of two Group I BldM targets, *sven1998* and *sven4150*. In both cases, BldM protected a region containing a well-conserved copy of the palindromic, MEME-predicted binding site (**Figure 2B**), consistent with this sequence serving as a high-affinity binding site for a BldM homodimer.

### Group II genes

Group II BldM target genes were expressed later than Group I genes (see insets in **Figure 1**). Further, and in contrast to Group I, the expression of Group II genes depended not only on *bldM* but also on *whiG* and *whiI* (**Figure 1** and **Table S2**). It is straightforward to account for the dependence of Group II gene expression on *whiG*. In *S. coelicolor*, *whiI* expression is activated from a single σ^WhiG^ target promoter, and thus *whiI* is not expressed in a *whiG* mutant [12]. This σ^WhiG^ target promoter appears well conserved at the sequence level in *S. venezuelae* (**Figure S2A**) and *whiI* is not expressed in an *S. venezuelae whiG* mutant (**Figure S2B**). Thus all genes that depend on *whiI* must necessarily also depend on *whiG*. Expression of *whiI* was not significantly affected in the *ΔbldM* mutant and *vice versa* (p<0.01) (**Figure S2B**), implying independent σ^WhiG^-WhiI and σ^BldN^-BldM regulatory pathways. The challenge then was to determine why Group II BldM target genes depend on *whiI*.

### Group II genes are direct *in vivo* targets of WhiI

**Figure 3:**
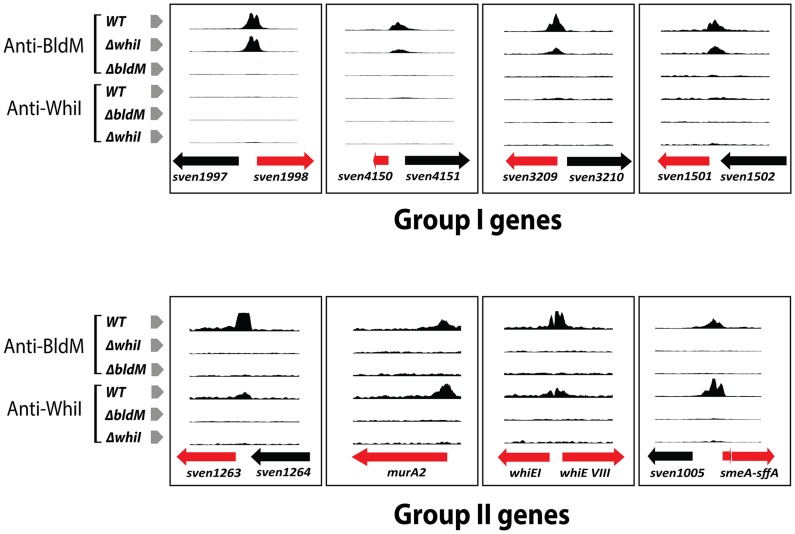
BldM and WhiI ChIP-Seq data for representative Group-I and Group-II promoters analysed in wild-type, *ΔbldM* and *ΔwhiI* backgrounds. Only BldM binds at Group-I promoters and this occurs both in the wild type and in the *ΔwhiI* mutant. Both BldM and WhiI bind at Group-II promoters in the wild type, but BldM binding depends on WhiI and *vice versa*. The genes shown in red are the targets genes.

To determine if the dependence of Group II BldM target genes on WhiI was direct or indirect, we characterized the *in vivo* WhiI binding sites across the *S. venezuelae* genome using ChIP-Seq. Wild-type *S. venezuelae* was harvested at 16 h of growth and treated as described for the BldM ChIP-Seq, except that a WhiI-specific polyclonal antibody was used. As a control, a ChIP-Seq experiment was performed using the congenic *whiI* null mutant. The data showed that all of the BldM Group II targets were also direct targets of WhiI (**Figure 3**). As an independent confirmation, we repeated the ChIP-Seq experiment using a FLAG-tagged WhiI protein. An N-terminally 3xFLAG-tagged allele of *whiI* (TF-WhiI) was constructed such that it was expressed from its native promoter and cloned into the single-copy vector pMS82, which integrates site-specifically into the chromosome at the phage ΦBT1 *attB* site [22]. This construct fully complemented the phenotype of the *whiI* null mutant (**Figure S3**), and the complemented strain was used for the ChIP-Seq experiment, now using wild-type *S. venezuelae* as the negative control. The ChIP-Seq results seen using FLAG immunoprecipitation were almost identical to those obtained using WhiI polyclonal antibodies, confirming that Group II genes are directly regulated by both BldM and WhiI (**Table S2**).

### At Group II promoters, *in vivo* WhiI binding depends on BldM and *vice versa*

Our data showed that expression of Group II genes depends on *bldM* and *whiI* and that both BldM and WhiI bind directly to the promoters of these genes. One possible model consistent with these observations would be that BldM and WhiI co-activate Group II promoters by binding as two separate homodimers. An alternative model would be that these two proteins activate Group II promoters by binding as a functional BldM-WhiI heterodimer. To begin to differentiate between these models, we performed BldM ChIP-Seq in a *ΔwhiI* mutant and WhiI ChIP-Seq in a *ΔbldM* mutant, using the same conditions described above. In a *ΔwhiI* mutant BldM binding was still observed at all Group I promoters (which depend solely on *bldM*), but no BldM binding was seen at Group II promoters (**Figure 3**). Equally, no WhiI binding to Group II promoters was observed in a *ΔbldM* mutant (**Figure 3**). Thus, *in vivo*, BldM and WhiI show mutual dependence for binding to Group II promoters.

### BldM and WhiI interact in *E. coli*

To explore the possibility of BldM-WhiI heterodimer formation, we tested BldM and WhiI for direct interaction in *E. coli* using a bacterial two-hybrid (BACTH) system [23]. *bldM* was fused to the gene encoding the T18 fragment of adenylate cyclase in the vector pUT18 such that BldM was at the N-terminus of the fusion protein, and was also fused to the gene encoding the T25 fragment of adenylate cyclase in the vector pKT25 such that BldM was at the C-terminus of the fusion protein. Parallel pUT18 and pKT25 constructs were made carrying fusions to WhiI.

**Figure 4:**
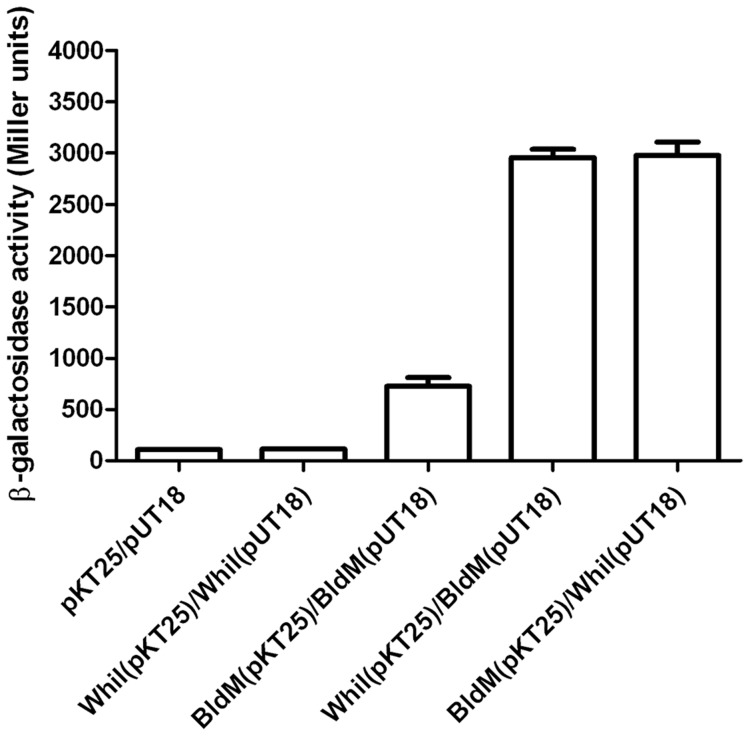
Bacterial two-hybrid analysis of BldM-WhiI interaction. The listed pairs of constructs were transferred into the BACTH reporter strain *E. coli* BTH101 by transformation. The resulting transformants were selected on MacConkey-maltose agar containing 100 µg/ml carbenicillin and 50 µg/ml kanamycin and incubated at 30°C for two days before four independent clones were picked and subjected to β-galactosidase assays. Error bars show the standard error of the four replicates carried out for each pairwise combination.

Interacting pairs of proteins were screened initially by transforming *E. coli* BTH101 with the appropriate plasmids and monitoring restoration of adenylate cyclase activity on X-gal indicator plates; clones of each pair were then assayed for β-galactosidase activity (**Figure 4**). Interaction of BldM with itself was readily observed (∼750 Miller units), but a stronger interaction (∼3000 Miller units), was observed between BldM and WhiI, regardless of which protein was fused to the T18 fragment of adenylate cyclase and which was fused to the T25 fragment (**Figure 4**). WhiI showed no detectable interaction with itself (**Figure 4**).

### BldM and WhiI interact in *Streptomyces*

To confirm and extend the BACTH analysis, we tested the interaction of BldM and WhiI in *Streptomyces* by coimmunoprecipitation. A C-terminally 3xFLAG-tagged allele of *bldM* (BldM-TF) was constructed such that it was expressed from its native promoter and cloned into the integrative vector pMS82 [22]. This construct complemented the phenotype of the *ΔbldM* null mutant to wild-type levels of sporulation (**Figure S3**). This strain was used in conjunction with the *ΔwhiI* mutant complemented with the N-terminally 3xFLAG-tagged allele of *whiI* (TF-WhiI) described above. The BldM-TF and TF-WhiI proteins were immunoprecipitated directly from 16 h MYM liquid cultures using the M2 anti-FLAG monoclonal antibody. WhiI coimmunoprecipitated with BldM-TF and BldM coimmunoprecipitated with TF-WhiI, but neither was detected in the negative controls when the wild-type strain was used (**Figure S4**). Thus BldM and WhiI interact in *Streptomyces in vivo*.

### Co-expression of BldM rescues WhiI from inclusion bodies

**Figure 5:**
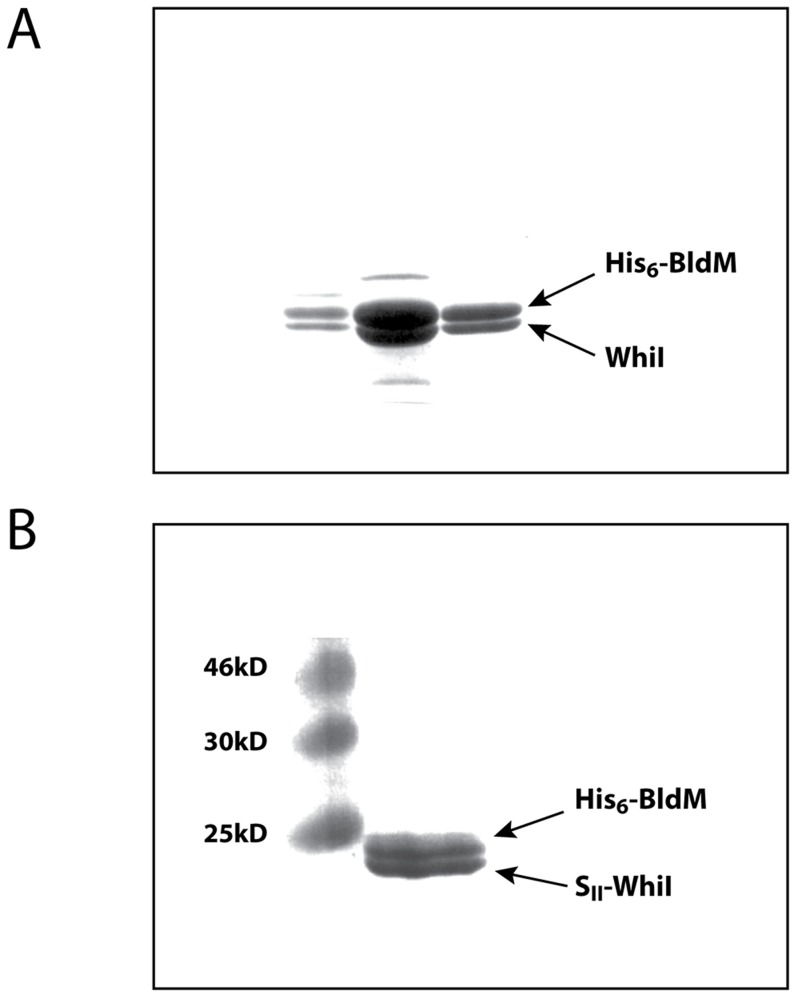
Affinity purification of the BldM-WhiI complex. N-terminally His_6_-tagged BldM and either (A) untagged WhiI, or (B) N-terminally S_II_-tagged WhiI, were co-expressed from the pETDuet-1 vector. In both cases, co-expression of BldM solubilized WhiI, which formed inclusion bodies when expressed alone. The His_6_-BldMWhiI complex in (A) was purified over a HisTrap Ni affinity column, and the His_6_-BldM-S_II_-whiI complex in (B) was purified over consecutive HisTrap Ni and StrepTrap HP affinity columns.

An early frustration in the *in vitro* analysis of WhiI function was that it overexpressed in an insoluble form in *E. coli* under all conditions tested. The realization that WhiI might act as part of a functional BldM-WhiI heterodimer led us to try an alternative approach. Where proteins form a complex, it is often observed that individual components are insoluble when expressed in isolation but become soluble when expressed with their cognate partner protein. Two examples are the α and β subunits of lambda integrase [24], and *Streptomyces* σ^BldN^ and its cognate anti-sigma factor, RsbN [16]. Accordingly, BldM and WhiI were co-expressed in *E. coli* using the pETDuet-1 system (Novagen). Initially, BldM was N-terminally His_6_-tagged and WhiI was left untagged. Co-expression of BldM was found to solubilize WhiI completely. Further, when His_6_-BldM was purified on a HisTrap Ni column, WhiI copurified with His_6_-BldM in approximately equal amounts, despite the fact that WhiI was untagged (**Figure 5A**) (the identity of the two proteins was confirmed by tryptic mass fingerprinting). Thus BldM rescues WhiI from inclusion bodies and the two proteins copurify in approximately stoichiometric amounts via a His_6_-tag present on BldM only.

This approach was extended by coexpressing BldM and WhiI carrying two compatible affinity tags. N-terminally His_6_-tagged BldM and N-terminally StrepII-tagged WhiI were co-expressed from the pETDuet-1 vector. As before, both BldM and WhiI were found in the soluble fraction. The BldM-WhiI complex was then purified over consecutive HisTrap Ni and StrepTrap HP affinity columns and the BldM and WhiI proteins were found to be present in stoichiometric amounts in the resulting preparation (**Figure 5B**).

### *In silico* analysis of Group II promoters

**Figure 6:**
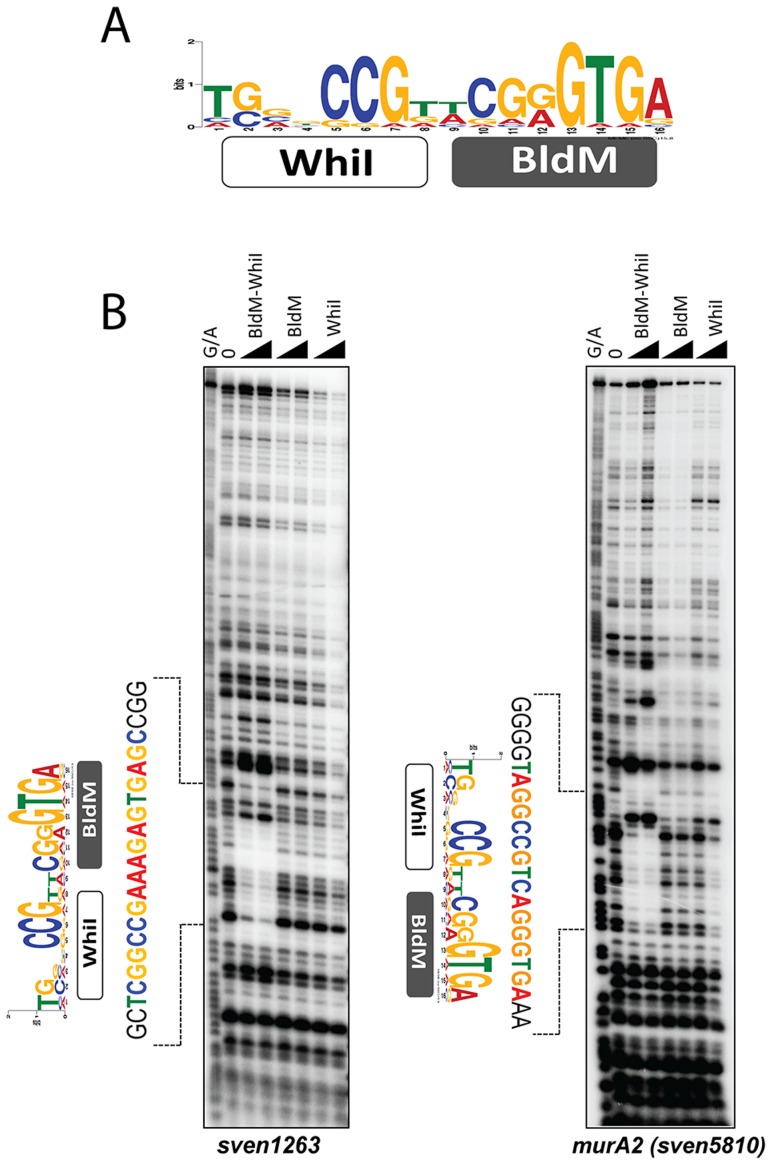
BldM-WhiI binds to the *MEME*-predicted Group-II consensus sequence. A. The *MEME*-predicted non-palindromic Group-II consensus sequence. The height of the letters in the sequence logo, in bits, is proportional to the frequency of the A, C, T, or G nucleotides at each position of the motif. B. DNase I footprinting analysis of BldM-WhiI, BldM and GST-WhiI binding to two Group-II promoters (*sven1263* and *murA2*). The protein concentrations used were 100 and 200 nM. G+A indicates the Maxam and Gilbert sequence ladder. The protected regions are indicated by dotted lines and the positions of the *MEME*-predicted binding motifs are shown.

To further understand Group II binding sites, we searched for over-represented sequences in Group II promoter regions using MEME [21], again using as input the entire intergenic region in each case. This analysis revealed a well-conserved 16 bp non-palindromic sequence, 5′-TGnnCCGnnCGGGTGA-3′, for which the sequence logo is shown in **Figure 6A**. Strikingly, the 3′ half of the Group II logo was equivalent to a half-site of the Group I palindrome, but the other half was different in sequence, potentially consistent with a BldM-WhiI heterodimer binding to Group II targets.

### BldM-WhiI binds to the MEME-predicted motifs in Group II promoters but BldM or GST-WhiI do not

To directly test the model that Group II promoters are controlled by a functional BldM-WhiI heterodimer, and to validate the MEME-predicted binding motif for these promoters, the doubly-tagged BldM-WhiI that had been purified over consecutive HisTrap Ni and StrepTrap HP affinity columns was used in DNase I footprinting analysis on the intergenic regions upstream of two Group II targets, *sven1263* and *murA2* (*sven5810*). BldM-WhiI footprinted on both promoters and in each case the protection region contained a copy of the non-palindromic, MEME-predicted binding site (**Figure 6B**), consistent with this sequence serving as a high-affinity binding site for a BldM-WhiI heterodimer. In contrast, neither BldM alone nor WhiI (produced as a soluble GST fusion), footprinted on either promoter (**Figure 6B**).

## Discussion

**Figure 7:**
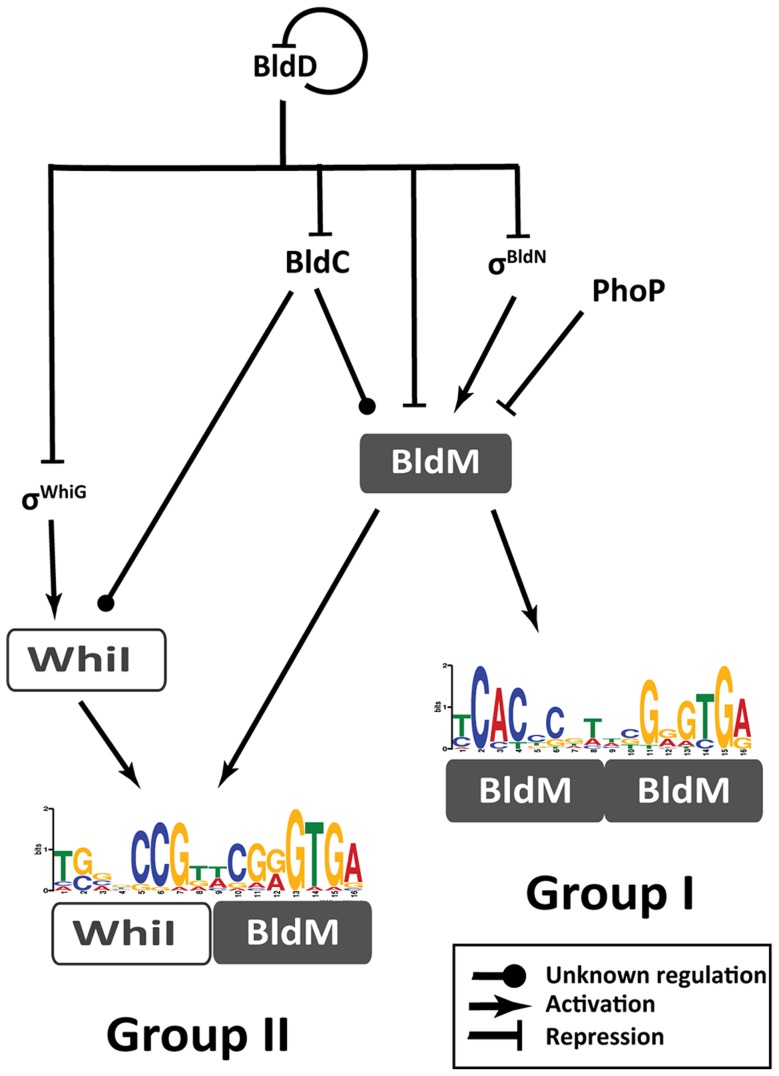
Schematic representation summarizing the regulatory network involved in controlling BldM and WhiI expression and the activation of Group-I and Group-II genes. During vegetative growth, BldD represses *bldC*, *bldN*, *whiG* and *bldM*. At the onset of differentiation, this repression is relieved, σ^BldN^ activates *bldM* and σ^WhiG^ activates *whiI*. BldM homodimer activates early sporulation genes such as *whiB* and *ssgR*. Subsequently, BldM and WhiI form a heterodimer that activates late sporulation genes such as *whiE* and *smeA-sffA*. Thus heterodimer formation serves to integrate signals from two independent pathways (σ^WhiG^-WhiI and σ^BldN^-BldM). *bldM* expression is tightly controlled by at least four global regulators - BldD, BldC, σ^BldN^ and PhoP (a response regulator activated during phosphate depletion [59]).

The focus of this study is to elucidate the mechanism underlying the direct co-activation by BldM and WhiI of the Group II genes, required for the late stages of development. Our data show that BldM activates transcription of these Group II genes as a BldM-WhiI heterodimer, while activating transcription of the Group I genes required for the early stages of development as a BldM homodimer. This work also significantly expands our knowledge of the regulatory network that controls morphological differentiation in *Streptomyces* (**Figure 7**), an advance made possible by exploiting *S. venezuelae* as a new model species for the genus.

### Group I genes

BldM homodimer activates several genes known to play key roles in the differentiation of aerial hyphae into spores, including *whiB* and *ssgR*. In addition to their positive regulation by BldM homodimer, these genes are also subject to repression during vegetative growth by the master regulator BldD, as is *bldM* itself (**Figure 7**) [25].

SsgA and SsgB are homologous proteins directly involved in the positive control of cell division in *Streptomyces* [26]. Sporogenic aerial hyphae undergo a synchronous round of cell division, initiated by the polymerization of a ladder of 50 or more FtsZ rings. SsgA and SsgB function in the recruitment and accurate positioning of these FtsZ rings, and *ΔssgA* and *ΔssgB* mutants of *S. coelicolor* lack sporulation septa [26]–[28]. *ssgR* encodes an IclR-family transcriptional regulator that directly activates the expression of *ssgA* in *S*. *coelicolor* [29]. Here we show that *ssgR* is directly activated by, and is completely dependent on BldM in *S. venezuelae*. *ssgB* is developmentally induced in *S. venezuelae*, but despite being a direct BldM target, its expression is only weakly affected in the *ΔbldM* mutant, suggesting complex regulation of this gene.

BldM homodimer also activates expression of *whiB*, which plays a vital role in developmentally controlled cell division. *whiB* null mutants fail to arrest aerial tip growth, the normal prelude to sporulation, and are completely blocked in the initiation of sporulation sepatation, producing abnormally long, undivided aerial hyphae [30]. WhiB is the founding member of a family of proteins confined to the actinomycetes, and several of these WhiB-like (Wbl) proteins have been shown to play key roles in the biology of streptomycetes and mycobacteria. Wbl proteins carry a [4Fe–4S] iron-sulfur cluster coordinated by four invariant cysteines in a C(X_29_)C(X_2_)C(X_5_)C motif [31]–[35], and although the biochemical role of these unusual proteins has been controversial [36], it seems increasingly certain that they function as transcription factors [33], [37]–[38].

### Group II genes

Among the Group II targets controlled by the BldM-WhiI heterodimer are two loci with well characterized roles in sporulation: *smeA-sffA* and *whiE*. The *smeA-sffA* operon encodes a DNA translocase (SffA) involved in chromosome segregation into spores that is specifically targeted to sporulation septa by the small membrane protein SmeA [39]. Deletion of *smeA-sffA* in *S. coelicolor* results in a defect in spore chromosome segregation and has pleiotropic effects on spore maturation [39]. Like the Group I targets *ssgR* and *whiB*, expression of the *smeA-sffA* operon is also repressed by the master regulator BldD during vegetative growth [25]. *whiE* is a complex locus that specifies the spore pigment. The structure of the spore pigment has not been determined in any *Streptomyces* species but its polyketide nature was first predicted from the sequence of the *whiE* locus in *S. coelicolor*, because it encodes proteins that closely resemble the components of type II polyketide synthases involved in the synthesis of aromatic antibiotics [40]–[42]. Based on their coordinate regulation and proposed functions, we predict the *whiE* locus of *S. venezuelae* consists of an operon of seven genes (*sven6798-6792*) and the divergently transcribed gene *sven6799*. Two distinct ChIP-Seq peaks were seen in the intergenic region separating *sven6799* and the *sven6798-6792* operon, and all eight genes fail to be expressed in the *bldM* and *whiI* mutants, implying the BldM-WhiI heterodimer controls expression of the entire locus.

### All WhiI-regulated genes belong to Group II

The work presented here suggests that there is no set of genes regulated by a WhiI homodimer and that WhiI functions as an auxiliary protein to modulate BldM binding specificity through heterodimerization. With no exceptions, all the genes down regulated in a *ΔwhiI* mutant were also down regulated in a *ΔbldM* mutant. Although some promoters were exclusively enriched as peaks in the WhiI ChIP-Seq experiment, without exception the transcriptional profile of such targets was unaffected in a *ΔwhiI* mutant, showing that WhiI has no regulatory influence on these genes. Further, in a *bldM* null mutant, some WhiI peaks are seen in ChIP-Seq, but these sites, often internal to ORFs, show no correlation with the wild-type WhiI regulon and the targets lacked a consensus binding motif. These results suggest that, in the absence of BldM, any DNA binding by WhiI is aberrant and unrelated to its behavior in the wild type. In contrast, in a *ΔwhiI* mutant, BldM fails to bind to Group II promoters but binds normally to its Group I promoters.

In a recent study, evidence was presented that the DNA-binding domain of *S. coelicolor* WhiI (in the absence of the receiver domain) can bind *in vitro* to the promoter of the *sco3900-sco3899* operon encoding a transcriptional regulator (InoR) and an inositol 1-phosphate synthase (InoA), respectively [43]. Our ChIP-Seq data show that WhiI does not regulate these genes in *S. venezuelae*. Further, in wild-type *S. venezuelae* both genes are actively expressed during vegetative growth but are down-regulated during development, and this expression pattern is unaffected in a *whiI* mutant.

### The potential of response regulator heterodimerization as a regulatory mechanism

Transcription factor heterodimerization can coordinate responses to different cues by integrating signals from distinct regulatory pathways. Although heterodimerization is prevalent as a regulatory mechanism in eukaryotes [44], it is rare in bacteria. Prior to the work described here, the only response regulator reported to heterodimerize with an auxiliary regulator was RcsB.

In *Escherichia coli*, the typical response regulator RcsB plays a central role in the regulation of capsule synthesis. Once phosphorylated by the histidine kinase RcsD, RcsB directly activates target genes including *rprA*, *osmC*, *osmB* and *ftsZ*, functioning as a homodimer [45], [46]. It also activates exopolysaccharide synthesis genes, required for capsule formation, as a heterodimer with RcsA, which is distantly related to response regulators (like RcsB, RcsA has a typical LuxR-type C-terminal DNA-binding domain, but its N-terminal domain is not related to typical response-regulator receiver domains). Like WhiI, RcsA appears to lack the capacity to activate genes by itself, and therefore functions solely as a modulator of RcsB binding specificity. RcsA is actively degraded by the Lon protease and in *lon* mutants capsule genes are highly upregulated causing a mucoid phenotype, due to enhanced activation by the stabilized RcsB-RcsA heterodimer [45], [46]. The capacity of RcsB to complex with other transcription factors is not limited to its interaction with RcsA, since RcsB also forms functional heterodimers with BglJ [47] and with GadE [48]. Using *in vitro* FRET analysis, weak hetero-pair interactions were detected between several members of the OmpR sub-family of response regulators from *E. coli* [49]. While these *in vitro* data may suggest potential crosstalk between distinct signaling pathways, their physiological significance has yet to be demonstrated.

The activities of numerous bacterial promoters respond to multiple cues, and there are many examples of promoters that depend on two activators for their activity. Several different regulatory mechanisms underpinning such codependence have been identified [50], [51]. The most widely documented is found at promoters where both activators bind independently, and both activators make independent contacts with RNA polymerase. However, there are rare examples of coactivators that exhibit cooperative binding, such as MelR and CRP at the *E. coli melAB* promoter [52]. There are also examples in which DNA binding by a secondary activator leads to the repositioning of the primary activator from a site where it cannot activate transcription to a site where it can, such as the repositioning of MalT by CRP at the *malK* promoter [53]. Response regulator heterodimer formation provides a new model for coactivation of target genes and the integration of regulatory signals at promoters. BldM-WhiI heterodimer formation serves to integrate signals from two independent pathways (σ^WhiG^-WhiI and σ^BldN^-BldM) and it may also function as a timing device, since Group II genes are activated later than Group I genes. Thus the BldM-WhiI system exemplifies the potential of response regulator heterodimer formation as a mechanism to expand the signaling capabilities of bacterial cells.

## Materials and Methods

### Bacterial strains, plasmids, oligonucleotides and growth conditions

Bacterial strains and plasmids are listed in **Table S3** and the oligonucleotide primers with corresponding restriction sites used in cloning are listed in **Table S4**. For microarray and ChIP-Seq experiments, *S*. *venezuelae* strains were grown at 30°C in MYM liquid sporulation medium [16] made with 50% tap water and supplemented with 200 µl trace element solution [54] per 100 ml. The phenotypes of mutants and complemented strains were scored after 3–4 days growth on MYM-agar at 30°C. *bldM* and *whiI* deletion mutants were constructed by ‘Redirect’ PCR targeting [55] and their chromosomal structures were confirmed by PCR analysis and by Southern hybridization using the parental cosmids as probes.

### ChIP (Chromatin immunoprecipitation)-Seq

For each strain, two flasks containing 35 ml of MYM were inoculated with spores (or mycelium in case of the *ΔbldM* mutant) to give an OD_600_ ∼0.35 after 8 h of growth. The crosslinking reagent formaldehyde was added to a final concentration of 1% (v/v) to the cultures at 16 h of growth and incubated at 30°C with shaking for 30 min before glycine was added to a final concentration of 125 mM to quench the crosslinking reaction. The samples were incubated at room temperature for 5 min and washed twice in PBS buffer pH 7.4 (Sigma). Mycelial pellets were resuspended in 0.5 ml lysis buffer (10 mM Tris-HCl pH 8, 50 mM NaCl, 15 mg/ml lysozyme, 1x protease inhibitor) and incubated at 37°C for 20 min. The lysate was resuspended in 0.5 ml IP buffer (100 mM Tris- HCl pH 8, 250 mM NaCl, 0.1% Triton-X-100, 1x protease inhibitor) and the lysate was kept on ice for 2 min before sonication. The samples were subjected to seven cycles of sonication, 15 s each, at 10 microns, to shear the chromosome into fragments ranging in size from 300–1000 bp. The sample was then centrifuged twice at top speed, 4°C for 15 minutes to clear cell extracts. To pre-clear non-specific binding, 90 µl protein A sepharose (Sigma) was added to cell lysate (about 900 µl) and incubated for 1 h at 4°C with mixing. The beads were cleared by centrifugation at top speed for 15 min. 100 µl BldM or WhiI antibodies were added to the corresponding cell lysates overnight at 4°C with mixing. 100 µl Protein A Sepharose 1∶1 suspension was added to immunoprecipitate antibody-BldM or WhiI chromatin complexes and incubated for 4 h at 4°C with mixing. The samples were centrifuged at 3500 rpm for 30 s and the beads were washed four times with IP buffer. The pellets were eluted in 150 µl IP elution buffer (50 mM Tris-HCl pH 7.6, 10 mM EDTA, 1% SDS) overnight at 65°C to reverse crosslink. The samples were centrifuged at top speed for 5 min to remove the beads and the pellets were re-extracted with 50 µl TE buffer (10 mM Tris-HCl pH 7.4, 1 mM EDTA). The supernatants were combined and incubated with 3 µl 10 mg/ml proteinase K (Roche) for 2 h at 55°C. The samples were extracted twice with phenol-chloroform to remove protein followed by chloroform extraction to remove traces of phenol and purified with Qiaquick columns (Qiagen). The IP DNA was eluted in 50 µl EB buffer (Qiagen). Sequencing libraries were generated and the IP DNA was sequenced as described previously [20]. The BayesPeak package was used to identify significantly enriched regions and the default parameters were applied [56].

### Microarray transcriptional profiling

Microarray transcriptional profiling experiments were carried out as described previously [16], [20]. Multi-experiment viewer software (MeV 4.8) was used for viewing and statistical analysis [57]. The non-parametric tool ‘Rank Products’ [58] was used in MeV to assign ‘down regulated’, ‘up regulated’ and ‘not significant’ genes based on expression at 16, 18 and 20 h of growth. Group I genes were defined as direct BldM ChIP-Seq targets that were significantly down regulated in *ΔbldM* and not significantly changed in all *Δwhi* mutants (**Table S1**). Group II genes were defined as direct ChIP-Seq targets of both BldM and WhiI that were significantly down regulated in *ΔbldM*, *ΔwhiG* and *ΔwhiI* (**Table S2**).

### Protein expression and purification

The open reading frame of interest was PCR-amplified using Expand High-Fidelity DNA polymerase (Roche). Plasmids containing the correct inserts were confirmed by sequencing and introduced into electrocompetent *E. coli* BL21(DE3)/pLysS. The transformed cells were spread on LB-carbenicillin/chloramphenicol and one colony was used for inoculation. Proteins were expressed in two 2.5 litre volumetric flasks each containing 400 ml LB culture and expression was induced with 0.25 mM IPTG. The optimised temperature for expression varied with the protein: His_6_-BldM was expressed at 25°C for 5 h; GST-WhiI was expressed at 15°C overnight; His_6_-BldM/WhiI or S_II_-WhiI were co-expressed at 30°C for 5 h. The pellets were lysed in a buffer containing 50 mM Tris-HCl pH 8, 250 mM NaCl, 10% glycerol, 0.1% Triton X100, protease inhibitor (complete mini, EDTA-free, Roche) and incubated at room temperature for 20 min. HisTrap HP Ni and StrepTrap HP affinity columns (GE Healthcare) were used to purify the His_6_- and S_II_-tagged proteins in a tandem manner. The Gst-WhiI was purified with 1 ml GSTrap FF column (GE Healthcare).

### DNase I footprinting

Singly ^32^P end-labelled probes (**Table S4**) were generated by PCR and purified using Qiaquick columns (Qiagen). Transcription factors were incubated with probe DNA (∼150,000 cpm) for 30 min at room temperature in 40 µl reaction buffer [50 mM Tris-HCl pH 7.5, 100 mM NaCl, 10% glycerol, 10 mM MgCl_2_, 2 mM dithiothreitol and 1 µg/reaction poly(dI-dC)], prior to treatment with 1 U DNase I (Promega) for 30–50 s in the case of group-II promoters and 3 U DNase I for 15–20 s in the case of group-I promoters. Reactions were terminated with 140 µl of stop buffer (192 mM sodium acetate, 32 mM EDTA, 0.14% SDS, 70 µg/ml yeast tRNA) and samples were extracted with phenol-chloroform prior to ethanol precipitation. Footprinting samples were loaded on 6% polyacrylamide sequencing gels, next to a G+A ladders prepared according to the Sure Track footprinting kit (Amersham Pharmacia Biotech).

### Immunoprecipitation of BldM-FLAG and FLAG-WhiI proteins from *S*. *venezuelae*

C-terminally 3×FLAG-tagged *bldM* expressed from the native promoter was used to complement the *ΔbldM* mutant and N-terminal 3×FLAG-tagged *whiI* carrying native promoter was used to complement the *ΔwhiI* mutant. 50 µl dense spore suspension was used to inoculate 300 ml MYM in 2 litre flasks with spring baffles. After 17 h growth, cultures were harvested by centrifugation at 6000 rpm for 15 min at 4°C. Pellets were lysed in a buffer containing 50 mM Tris-HCl pH 8, 250 mM NaCl, 10% glycerol, 0.1% Triton X100, protease inhibitor (complete mini, EDTA-free, Roche) and sonicated for 5 cycles at 15-micron amplitude for 20 s. FLAG-tagged proteins were immunoprecipitiated using M2 beads [anti-FLAG antibodies covalently attached to agarose beads (Sigma)], the beads were washed using TBS buffer containing protease inhibitor (Roche) and 0.05% Triton X100, and the protein was eluted using FLAG peptides as recommended by the manufacturer.

### Preparation of crude cell extracts and immunoblot analysis

*S*. *venezuelae* strains were grown in MYM medium. 10 ml samples were taken at 14, 15, and 16 hours of growth. Strains were harvested by centrifugation at 3000 rpm for 1 min, washed in 5 ml ice-cold washing buffer (20 mM Tris pH 8.0, 5 mM EDTA) and resuspended in 0.4 ml of ice-cold sonication buffer [20 mM Tris pH 8.0, 5 mM EDTA, 1x protease inhibitor (Roche)]. Samples were sonicated immediately for 5 cycles, 20 s at 10 microns with 1 min intervals of ice incubation, then centrifuged at 13000 rpm at 4°C for 15 min to remove cell debris. Protein concentrations of the supernatant crude cell extracts were measured by Bradford assay and samples (10 µg protein) were separated on a 12.5% SDS-PA gel and blotted onto nitrocellulose membrane. The membrane was incubated in blocking solution [10% dried milk powder in TBS (0.05 M Tris, 0.9% NaCl, pH 7.6, 0.1% Tween)] overnight and then incubated for 1 h at room temperature with the 1/2500 dilution of anti-BldM antiserum in blocking solution. The membrane was rinsed (twice for 10 min) in TBS and then incubated for 1 h with 1/5,000 dilutions of horseradish peroxidase-linked goat anti-rabbit immunoglobulin G antibody (GE Healthcare). Blots were developed using the ECL enhanced chemiluminescence system from GE Healthcare and were typically exposed to X- ray film for between 30 s and 5 min.

## Supporting Information

Figure S1 Western blot analysis of BldM levels during differentiation of wild-type *S. venezuelae* grown in MYM liquid sporulation medium. Polyclonal anti-BldM antibodies were used.
(TIF)

Figure S2 **A**. Alignment of the *whiI* promoters of *S. coelicolor* and *S. venezuelae* showing conservation of the σ^WhiG^ -10 and -35 sequences. **B**. Transcriptional profiles of *bldM* and *whiI* during differentiation of wild-type *S. venezuelae* (WT) and its congenic *bldM*, *whiG* and *whiI* mutants. *bldM* transcript levels are indicated in blue and *whiI* transcript levels are indicated in red. Note that transcription of *bldM* and *whiI* cannot be detected in their respective null mutants because the coding sequences represented on the microarrays are deleted in those strains. Strains were grown in MYM liquid sporulation medium.
(TIF)

Figure S3 **A**. Complementation of the *ΔbldM* mutant with a C-terminal *bldM-3xFLAG* allele cloned into the integrative vector pMS82. **B**. Complementation of the *ΔwhiI* mutant with an N-terminal *3xFLAG-whiI* allele cloned into the integrative vector pMS82.
(TIF)

Figure S4 Co-immunoprecipitation of BldM and WhiI. **A**. The *ΔwhiI* mutant complemented with the N-terminally 3xFLAG-tagged allele of *whiI* was grown for 16 h in MYM liquid sporulation medium, and FLAG-WhiI was immunoprecipitated using M2 antibody. Polyclonal BldM antibody was used to detect the presence of BldM, and immunoprecipitates from WT and BldM-FLAG strains were used as negative and positive controls, respectively. **B**. The *ΔbldM* mutant complemented with the C-terminally 3xFLAG-tagged allele of *bldM* was grown for 16 h in MYM liquid sporulation medium and BldM-FLAG was immunoprecipitated as described above. Polyclonal WhiI antibody was used to detect the presence of WhiI, and immunoprecipitates from WT and FLAG-WhiI strains were used as negative and positive controls, respectively.
(TIF)

Table S1 List of Group I genes describing peak positions, annotations, microarray fold change and p values for expression in the *ΔbldM* and *ΔwhiI* mutant backgrounds.
(XLSX)

Table S2 List of Group II genes describing peak positions, annotations, microarray fold change and p values for expression in the *ΔbldM* and *ΔwhiI* mutant backgrounds. The presence or absence of peaks in the corresponding FLAG-WhiI ChIP-Seq experiment is also shown.
(XLSX)

Table S3 Strains and plasmids used in this study.
(DOCX)

Table S4 Oligonucleotides used for cloning and DNase I footprinting. Restriction sites in the primers are shown.
(XLSX)

## References

[1] Capra EJ, Laub MT (2012) Evolution of two-component signal transduction systems. Annu Rev Microbiol 66: 325–347. 22746333 10.1146/annurev-micro-092611-150039 PMC4097194

[2] Gao R, Stock AM (2009) Biological insights from structures of two-component proteins. Annu Rev Microbiol 63: 133–154. 19575571 10.1146/annurev.micro.091208.073214 PMC3645274

[3] Mitrophanov AY, Groisman EA (2008) Signal integration in bacterial two-component regulatory systems. Genes Dev 22: 2601–2611. 18832064 10.1101/gad.1700308 PMC2751022

[4] Elliot MA, Buttner MJ, Nodwell JR (2008) Multicellular Development in *Streptomyces* In: Whitworth DE, editor. *Myxobacteria*: Multicellularity and Differentiation. Washington D.C.: ASM Press. pp. 419–438.

[5] Flärdh K, Buttner MJ (2009) *Streptomyces* morphogenetics: dissecting differentiation in a filamentous bacterium. Nat Rev Microbiol 7: 36–49. 19079351 10.1038/nrmicro1968

[6] McCormick JR, Flärdh K (2012) Signals and regulators that govern *Streptomyces* development. FEMS Microbiol Rev 36: 206–231. 22092088 10.1111/j.1574-6976.2011.00317.x PMC3285474

[7] Bourret RB (2010) Receiver domain structure and function in response regulator proteins. Curr Opin Microbiol 13: 142–149. 20211578 10.1016/j.mib.2010.01.015 PMC2847656

[8] Hong E, Lee HM, Ko H, Kim DU, Jeon BY, et al (2007) Structure of an atypical orphan response regulator protein supports a new phosphorylation-independent regulatory mechanism. J Biol Chem 282: 20667–20675. 17491010 10.1074/jbc.M609104200

[9] Fraser JS, Merlie JP Jr, Echols N, Weisfield SR, Mignot T, et al (2007) An atypical receiver domain controls the dynamic polar localization of the *Myxococcus xanthus* social motility protein FrzS. Mol Microbiol 65: 319–332. 17573816 10.1111/j.1365-2958.2007.05785.x PMC1974792

[10] Wang L, Tian X, Wang J, Yang H, Fan K, et al (2009) Autoregulation of antibiotic biosynthesis by binding of the end product to an atypical response regulator. Proc Natl Acad Sci U S A 106: 8617–8622. 19423672 10.1073/pnas.0900592106 PMC2688989

[11] Hickey JM, Lovell S, Battaile KP, Hu L, Middaugh CR, et al (2011) The atypical response regulator protein ChxR has structural characteristics and dimer interface interactions that are unique within the OmpR/PhoB subfamily. J Biol Chem 286: 32606–32616. 21775428 10.1074/jbc.M111.220574 PMC3173177

[12] Aínsa JA, Parry HD, Chater KF (1999) A response regulator-like protein that functions at an intermediate stage of sporulation in *Streptomyces coelicolor* A3(2). Mol Microbiol 34: 607–619. 10564501 10.1046/j.1365-2958.1999.01630.x

[13] Tian Y, Fowler K, Findlay K, Tan H, Chater KF (2007) An unusual response regulator influences sporulation at early and late stages in *Streptomyces coelicolor* . J Bacteriol 189: 2873–2885. 17220225 10.1128/JB.01615-06 PMC1855786

[14] Molle V, Buttner MJ (2000) Different alleles of the response regulator gene *bldM* arrest *Streptomyces coelicolor* development at distinct stages. Mol Microbiol 36: 1265–1278. 10931278 10.1046/j.1365-2958.2000.01977.x

[15] Bibb MJ, Molle V, Buttner MJ (2000) σ^BldN^, an extracytoplasmic function RNA polymerase sigma factor required for aerial mycelium formation in *Streptomyces coelicolor* A3(2). J Bacteriol 182: 4606–4616. 10913095 10.1128/jb.182.16.4606-4616.2000 PMC94633

[16] Bibb MJ, Domonkos Á, Chandra G, Buttner MJ (2012) Expression of the chaplin and rodlin hydrophobic sheath proteins in *Streptomyces venezuelae* is controlled by σ^BldN^ and a cognate anti-sigma factor, RsbN. Mol Microbiol 84: 1033–1049. 22582857 10.1111/j.1365-2958.2012.08070.x

[17] Elliot MA, Karoonuthaisiri N, Huang J, Bibb MJ, Cohen SN, et al (2003) The chaplins: a family of hydrophobic cell-surface proteins involved in aerial mycelium formation in *Streptomyces coelicolor* . Genes Dev 17: 1727–1740. 12832397 10.1101/gad.264403 PMC196181

[18] Claessen D, Rink R, de Jong W, Siebring J, de Vreugd P, et al (2003) A novel class of secreted hydrophobic proteins is involved in aerial hyphae formation in *Streptomyces coelicolor* by forming amyloid-like fibrils. Genes Dev 17: 1714–1726. 12832396 10.1101/gad.264303 PMC196180

[19] Claessen D, Stokroos I, Deelstra HJ, Penninga NA, Bormann C, et al (2004) The formation of the rodlet layer of streptomycetes is the result of the interplay between rodlins and chaplins. Mol Microbiol 53: 433–443. 15228525 10.1111/j.1365-2958.2004.04143.x

[20] Bush MJ, Bibb MJ, Chandra G, Findlay KC, Buttner MJ (2013) Genes required for aerial growth, cell division, and chromosome segregation are targets of WhiA before sporulation in *Streptomyces venezuelae* . MBio 4: e00684–13. 24065632 10.1128/mBio.00684-13 PMC3781837

[21] Bailey TL, Elkan C (1994) Fitting a mixture model by expectation maximization to discover motifs in biopolymers. In: Proceedings of the Second International Conference on Intelligent Systems for Molecular Biology. Menlo Park: AAAI Press. pp 28–36. 7584402

[22] Gregory MA, Till R, Smith MCM (2003) Integration site for *Streptomyces* phage ΦBT1 and development of site-specific integrating vectors. J Bacteriol 185: 5320–5323. 12923110 10.1128/JB.185.17.5320-5323.2003 PMC180994

[23] Karimova G, Pidoux J, Ullmann A, Ladant D (1998) A bacterial two-hybrid system based on a reconstituted signal transduction pathway. Proc Natl Acad Sci USA 95: 5752–5756. 9576956 10.1073/pnas.95.10.5752 PMC20451

[24] Nash HA, Robertson CA, Flamm E, Weisberg RA, Miller HI (1987) Overproduction of *Escherichia coli* integration host factor, a protein with nonidentical subunits. J Bacteriol 169: 4124–4127. 3305480 10.1128/jb.169.9.4124-4127.1987 PMC213718

[25] den Hengst CD, Tran NT, Bibb MJ, Chandra G, Leskiw BK, et al (2010) Genes essential for morphological development and antibiotic production in *Streptomyces coelicolor* are targets of BldD during vegetative growth. Mol Microbiol 78: 361–379. 20979333 10.1111/j.1365-2958.2010.07338.x

[26] Willemse J, Borst JW, de Waal E, Bisseling T, van Wezel GP (2011) Positive control of cell division: FtsZ is recruited by SsgB during sporulation of *Streptomyces* . Genes Dev 25: 89–99. 21205868 10.1101/gad.600211 PMC3012939

[27] van Wezel GP, van der Meulen J, Kawamoto S, Luiten RG, Koerten HK, et al (2000) *ssgA* is essential for sporulation of *Streptomyces coelicolor* A3(2) and affects hyphal development by stimulating septum formation. J Bacteriol 182: 5653–5662. 11004161 10.1128/jb.182.20.5653-5662.2000 PMC94684

[28] Keijser BJ, Noens EE, Kraal B, Koerten HK, van Wezel GP (2003) The *Streptomyces coelicolor ssgB* gene is required for early stages of sporulation. FEMS Microbiol Lett 225: 59–67. 12900022 10.1016/S0378-1097(03)00481-6

[29] Traag BA, Kelemen GH, van Wezel GP (2004) Transcription of the sporulation gene *ssgA* is activated by the IclR-type regulator SsgR in a *whi*-independent manner in *Streptomyces coelicolor* A3(2). Mol Microbiol 53: 985–1000. 15255907 10.1111/j.1365-2958.2004.04186.x

[30] Flärdh K, Findlay KC, Chater KF (1999) Association of early sporulation genes with suggested developmental decision points in *Streptomyces coelicolor* A3(2). Microbiology 145: 2229–2243. 10517576 10.1099/00221287-145-9-2229

[31] Jakimowicz P, Cheesman MR, Bishai WR, Chater KF, Thomson AJ, et al (2005) Evidence that the Streptomyces developmental protein WhiD, a member of the WhiB family, binds a [4Fe-4S] cluster. J Biol Chem 280: 8309–8315. 15615709 10.1074/jbc.M412622200

[32] Singh A, Guidry L, Narasimhulu KV, Mai D, Trombley J, et al (2007) *Mycobacterium tuberculosis* WhiB3 responds to O_2_ and nitric oxide via its [4Fe-4S] cluster and is essential for nutrient starvation survival. Proc Natl Acad Sci U S A 104: 11562–11567. 17609386 10.1073/pnas.0700490104 PMC1906726

[33] Singh A, Crossman DK, Mai D, Guidry L, Voskuil MI, et al (2009) *Mycobacterium tuberculosis* WhiB3 maintains redox homeostasis by regulating virulence lipid anabolism to modulate macrophage response. PLoS Pathog 5: e1000545. 19680450 10.1371/journal.ppat.1000545 PMC2718811

[34] Crack JC, den Hengst CD, Jakimowicz P, Subramanian S, Johnson MK, et al (2009) Characterization of [4Fe-4S]-containing and cluster-free forms of *Streptomyces* WhiD. Biochemistry 48: 12252–12264. 19954209 10.1021/bi901498v PMC2815329

[35] Crack JC, Smith LJ, Stapleton MR, Peck J, Watmough NJ, et al (2011) Mechanistic insight into the nitrosylation of the [4Fe-4S] cluster of WhiB-like proteins. J Am Chem Soc 133: 1112–1121. 21182249 10.1021/ja109581t PMC3117330

[36] den Hengst CD, Buttner MJ (2008) Redox control in actinobacteria. Biochem Biophys Acta 1780: 1201–1216. 18252205 10.1016/j.bbagen.2008.01.008

[37] Smith L, Stapleton MR, Fullstone GJ, Crack JC, Thomson AJ, et al (2010) *Mycobacterium tuberculosis* WhiB1 is an essential DNA-binding protein with a nitric oxide-sensitive iron-sulfur cluster. Biochem J 432: 417–427. 20929442 10.1042/BJ20101440 PMC2992795

[38] Rybniker J, Nowag A, van Gumpel E, Nissen N, Robinson N, et al (2010) Insights into the function of the WhiB-like protein of mycobacteriophage TM4 - a transcriptional inhibitor of WhiB2. Mol Microbiol 77: 642–657. 20545868 10.1111/j.1365-2958.2010.07235.x

[39] Ausmees N, Wahlstedt H, Bagchi S, Elliot MA, Buttner MJ, et al (2007) SmeA, a small membrane protein with multiple functions in *Streptomyces sporulation* including targeting of a SpoIIIE/FtsK-like protein to cell division septa. Mol Microbiol 65: 1458–1473. 17824926 10.1111/j.1365-2958.2007.05877.x

[40] Davis NK, Chater KF (1990) Spore colour in *Streptomyces coelicolor* A3(2) involves the developmentally regulated synthesis of a compound biosynthetically related to polyketide antibiotics. Mol Microbiol 4: 1679–1691. 2077356 10.1111/j.1365-2958.1990.tb00545.x

[41] Yu T-W, Hopwood DA (1995) Ectopic expression of the *Streptomyces coelicolor whiE* genes for polyketide spore pigment synthesis and their interaction with the *act* genes for actinorhodin biosynthesis. Microbiology 141: 2779–2791. 8535506 10.1099/13500872-141-11-2779

[42] Kelemen GH, Brian P, Flärdh K, Chamberlin L, Chater KF, et al (1998) Developmental regulation of transcription of *whiE*, a locus specifying the polyketide spore pigment in *Streptomyces coelicolor* A3(2). J Bacteriol 180: 2515–2521. 9573206 10.1128/jb.180.9.2515-2521.1998 PMC107196

[43] Zhang G, Tian Y, Hu K, Zhu Y, Chater KF, et al (2012) Importance and regulation of inositol biosynthesis during growth and differentiation of *Streptomyces* . Mol Microbiol 83: 1178–1194. 22329904 10.1111/j.1365-2958.2012.08000.x

[44] Reményi A, Schöler HR, Wilmanns M (2004) Combinatorial control of gene expression. Nat Struct Mol Biol 11: 812–815. 15332082 10.1038/nsmb820

[45] Majdalani N, Gottesman S (2005) The Rcs phosphorelay: a complex signal transduction system. Annu Rev Microbiol 59: 379–405. 16153174 10.1146/annurev.micro.59.050405.101230

[46] Majdalani N, Gottesman S (2007) Genetic dissection of signaling through the Rcs phosphorelay. Methods Enzymol 423: 349–362. 17609140 10.1016/S0076-6879(07)23016-2

[47] Venkatesh GR, Kembou Koungni FC, Paukner A, Stratmann T, Blissenbach B, et al (2010) BglJ-RcsB heterodimers relieve repression of the *Escherichia coli bgl* operon by H-NS. J Bacteriol 192: 6456–6464. 20952573 10.1128/JB.00807-10 PMC3008536

[48] Castanié-Cornet MP, Cam K, Bastiat B, Cros A, Bordes P, et al (2010) Acid stress response in *Escherichia coli*: mechanism of regulation of *gadA* transcription by RcsB and GadE. Nucleic Acids Res 38: 3546–3554. 20189963 10.1093/nar/gkq097 PMC2887963

[49] Gao R, Tao Y, Stock AM (2008) System-level mapping of *Escherichia coli* response regulator dimerization with FRET hybrids. Mol Microbiol 69: 1358–1372. 18631241 10.1111/j.1365-2958.2008.06355.x PMC2586830

[50] Lee DJ, Minchin SD, Busby SJ (2012) Activating transcription in bacteria. Annu Rev Microbiol 66: 125–152. 22726217 10.1146/annurev-micro-092611-150012

[51] Browning DF, Busby SJ (2004) The regulation of bacterial transcription initiation. Nat Rev Microbiol 2: 57–65. 15035009 10.1038/nrmicro787

[52] Wade JT, Belyaeva TA, Hyde EI, Busby SJ (2001) A simple mechanism for co-dependence on two activators at an *Escherichia coli* promoter. EMBO J 20: 7160–7167. 11742992 10.1093/emboj/20.24.7160 PMC125794

[53] Richet E, Vidal-Ingigliardi D, Raibaud O (1991) A new mechanism for coactivation of transcription initiation: repositioning of an activator triggered by the binding of a second activator. Cell 66: 1185–1195. 1913806 10.1016/0092-8674(91)90041-v

[54] Kieser T, Bibb MJ, Buttner MJ, Chater KF, Hopwood DA (2000) Practical *Streptomyces* Genetics. The John Innes Foundation. Norwich, United Kingdom.

[55] Gust B, Challis GL, Fowler K, Kieser T, Chater KF (2003) PCR-targeted *Streptomyces* gene replacement identifies a protein domain needed for biosynthesis of the sesquiterpene soil odor geosmin. Proc Natl Acad Sci U S A 100: 1541–1546. 12563033 10.1073/pnas.0337542100 PMC149868

[56] Cairns J, Spyrou C, Stark R, Smith ML, Lynch AG, et al. 2011. BayesPeak—an R package for analysing ChIP-seq data. Bioinformatics 27: 713–714. 21245054 10.1093/bioinformatics/btq685 PMC3042177

[57] Saeed AI, Bhagabati NK, Braisted JC, Liang W, Sharov V, et al (2006) TM4 microarray software suite. Methods Enzymol 411: 134–193. 16939790 10.1016/S0076-6879(06)11009-5

[58] Breitling R, Armengaud P, Amtmann A, Herzyk P (2004) Rank products: a simple, yet powerful, new method to detect differentially regulated genes in replicated microarray experiments. FEBS Lett 573: 83–92. 15327980 10.1016/j.febslet.2004.07.055

[59] Allenby NE, Laing E, Bucca G, Kierzek AM, Smith CP (2012) Diverse control of metabolism and other cellular processes in *Streptomyces coelicolor* by the PhoP transcription factor: genome-wide identification of *in vivo* targets. Nucleic Acids Res 40: 9543–9556. 22904076 10.1093/nar/gks766 PMC3479208

