# Supplementary figures and images for: Response Regulator Heterodimer Formation Controls a Key Stage in Streptomyces Development
Source: PLoS Genet. 2014 Aug 7;10(8):e1004554. doi: 10.1371/journal.pgen.1004554 (PMC4125116; doi:10.1371/journal.pgen.1004554)

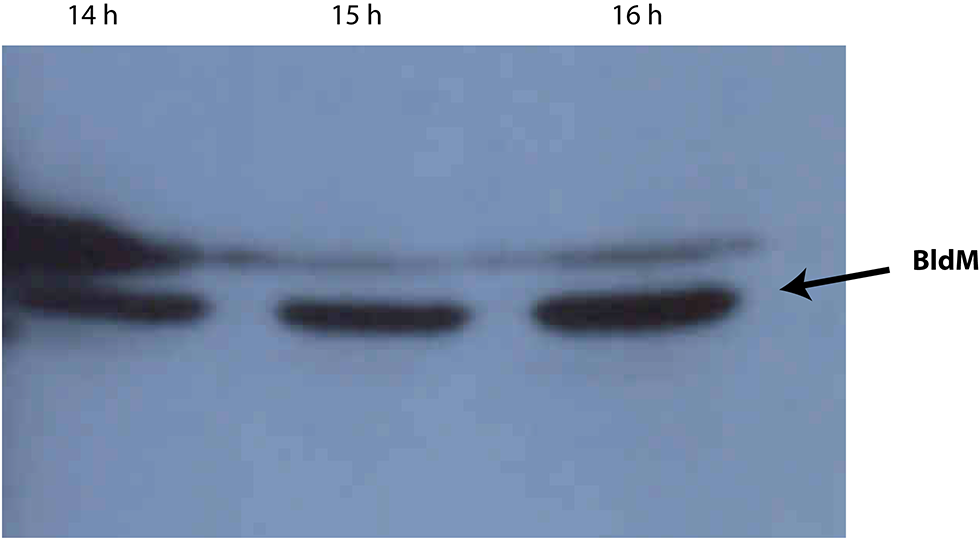

Supplement: Figure S1 — Western blot analysis of BldM levels during differentiation of wild-type S. venezuelae grown in MYM liquid sporulation medium. Polyclonal anti-BldM antibodies were used. (TIF) [file pgen.1004554.s001.tif]

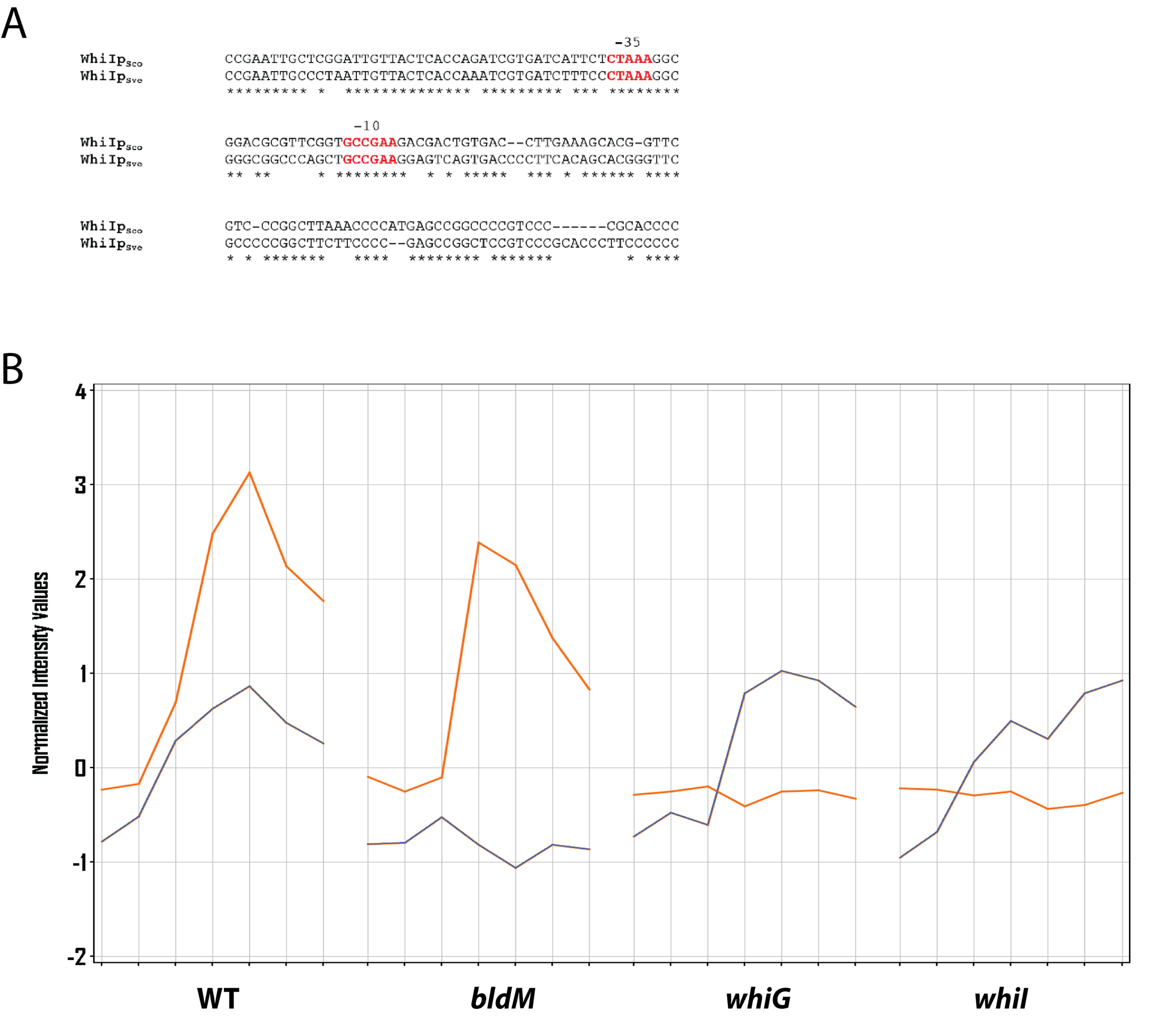

Supplement: Figure S2 — A. Alignment of the whiI promoters of S. coelicolor and S. venezuelae showing conservation of the σWhiG -10 and -35 sequences. B. Transcriptional profiles of bldM and whiI during differentiation of wild-type S. venezuelae (WT) and its congenic bldM, whiG and whiI mutants. bldM transcript levels are indicated in blue and whiI transcript levels are indicated in red. Note that transcription of bldM and whiI cannot be detected in their respective null mutants because the coding sequences represented on the microarrays are deleted in those strains. Strains were grown in MYM liquid sporulation medium. (TIF) [file pgen.1004554.s002.tif]

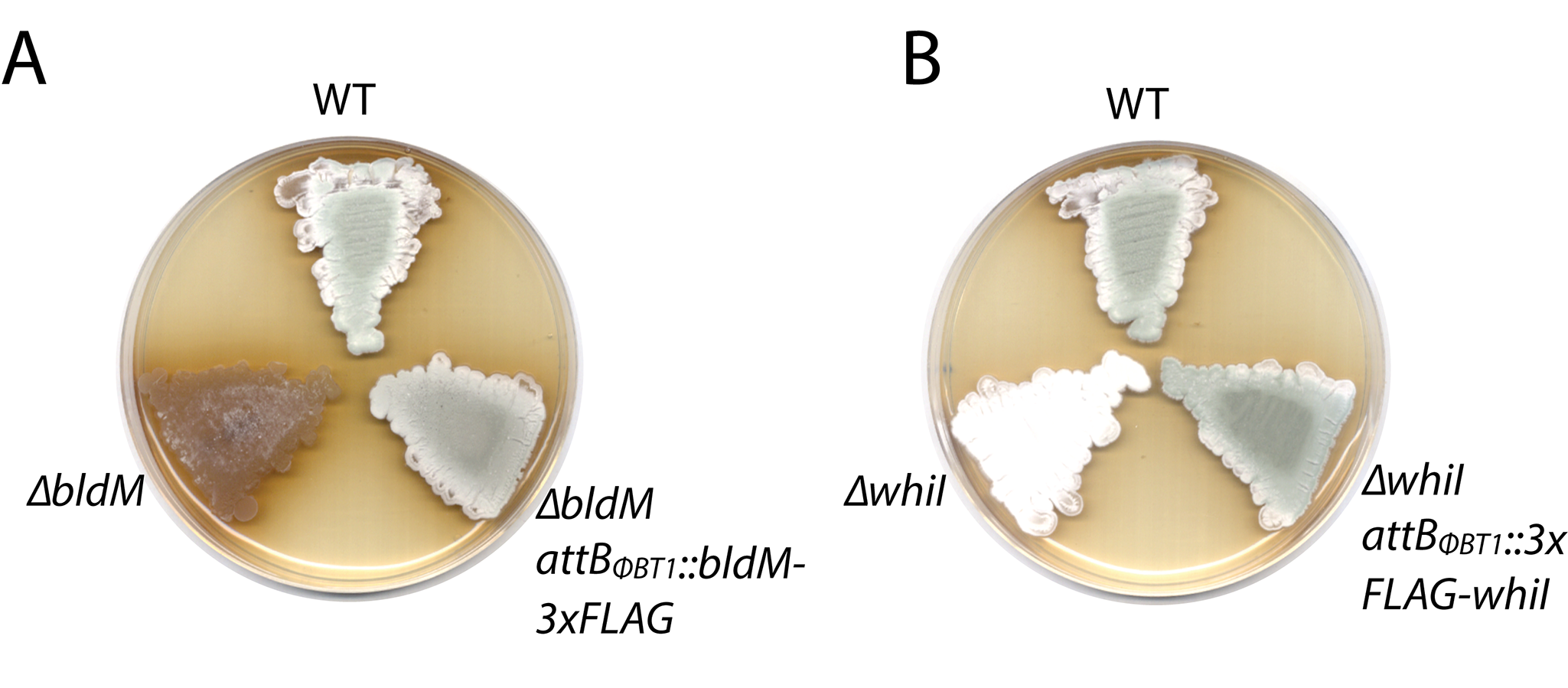

Supplement: Figure S3 — A. Complementation of the ΔbldM mutant with a C-terminal bldM-3xFLAG allele cloned into the integrative vector pMS82. B. Complementation of the ΔwhiI mutant with an N-terminal 3xFLAG-whiI allele cloned into the integrative vector pMS82. (TIF) [file pgen.1004554.s003.tif]

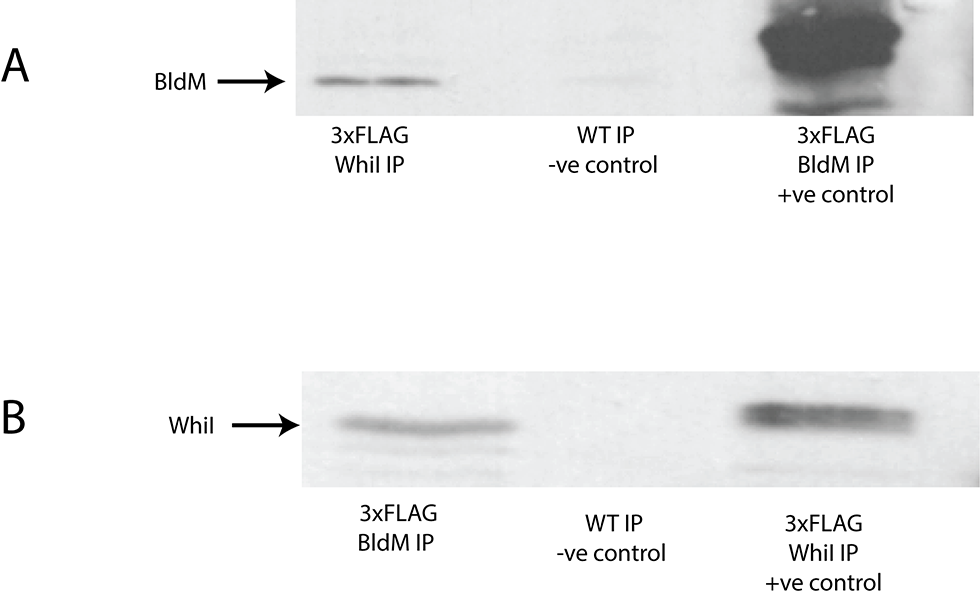

Supplement: Figure S4 — Co-immunoprecipitation of BldM and WhiI. A. The ΔwhiI mutant complemented with the N-terminally 3xFLAG-tagged allele of whiI was grown for 16 h in MYM liquid sporulation medium, and FLAG-WhiI was immunoprecipitated using M2 antibody. Polyclonal BldM antibody was used to detect the presence of BldM, and immunoprecipitates from WT and BldM-FLAG strains were used as negative and positive controls, respectively. B. The ΔbldM mutant complemented with the C-terminally 3xFLAG-tagged allele of bldM was grown for 16 h in MYM liquid sporulation medium and BldM-FLAG was immunoprecipitated as described above. Polyclonal WhiI antibody was used to detect the presence of WhiI, and immunoprecipitates from WT and FLAG-WhiI strains were used as negative and positive controls, respectively. (TIF) [file pgen.1004554.s004.tif]
