# Supplementary material for: Response Regulator Heterodimer Formation Controls a Key Stage in Streptomyces Development
Source: PLoS Genet. 2014 Aug 7;10(8):e1004554. doi: 10.1371/journal.pgen.1004554 (PMC4125116; doi:10.1371/journal.pgen.1004554)
Supplement: Table S3 — Strains and plasmids used in this study. (DOCX) [file pgen.1004554.s007.docx]

**Table S3. Bacterial strains and plasmids**

Strain/plasmid Genotype Source/Reference

***E. coli***

ET12567 F^–^ *dam13::Tn9 dcm6 hsdM hsdR* MacNeil *et al*. (1992)

*recF143::Tn10 galK2 galT22 ara-14*

*lacY1 xyl-5 leuB6 thi-1 tonA31 rpsL*

*hisG4 tsx-78 mtl-1 glnV44*

ET12567 ET12567 containing helper plasmid Paget *et al*. (1999)

pUZ8002

BW25113 *Δ*(*araD-araB*)*567 ΔlacZ4787*(*::rrnB-4*) Datsenko & Wanner,

*lacIp-4000*(*lacI^Q^*) *λ-rpoS369*(*Am*) *rph-1* (2000)

*Δ*(*rhaD-rhaB*)*568 hsdR514*

BL21 (DE3)/ F^–^ *omp*T *gal dcm lon hsd*S_B_(r_B_^-^ m_B_^-^) Studier & Moffatt

pLysS λ(DE3 [*lac*I *lacUV5*-T7 gene 1 *ind*1 (1986)

*sam*7 *nin*5]) pLysS (Cam^R^)

BTH101 F^–^ *cya-99 araD139 galE15 galK16* Karimova *et al*.

*rpsL1* *(Str^r^) hsdR2 mcrA1 mcrB1* (2000)

***S. venezuelae***

ATCC10712 wild-type *S. venezuelae* Ehrlich *et al*. (1948)

SV10 Δ*whiI::apr-oriT*  This work

SV13 Δ*bldM::apr-oriT* This work

**Plasmids**

pIJ773 pBluescript KS (+) containing the Gust *et al*. (2003)

apramycin resistance gene *apr* and

*oriT* of plasmid RP4, flanked by

FRT sites (Apr^R^)

pIJ790 Modified λ RED recombination Gust *et al*. (2003)

plasmid pKD20 (Cam^R^)

pUC19 *E. coli* multicopy cloning vector with Yanisch -Perron *et al*.

*lacZ* screening (Bla^R^) (1985)

pDrive *E. coli* multicopy cloning vector with Qiagen

*lacZ* screening (Bla^R^  Neo^R^)

pUZ8002 Non-transmissible *oriT*-mobilising Paget *et al*. (1999)

plasmid (Kan^R^)

pET15b N-terminal His_6_-tagging, IPTG- Novagen

inducible protein expression vector

pETDuet-1 co-expression vector; N-terminal His- Novagen

tagging and C-terminal S-tagging

pIJ6613 pET15b carrying wild-type *bldM* Molle & Buttner (2000)

ORF (Bla^R^)

pIJ10428 pET15b carrying *whiIsve* ORF This work

pMS82 Plasmid cloning vector for the Gregory *et al*. (2003)

conjugal transfer of DNA from

*E. coli* to *Streptomyces*

integrates site-specifically at the

ΦBT1 attachment site (Hyg^R^)

pIJ10429 pMS82 carrying *bldM* ORF with This work

C-terminal triple FLAG with native

promoter (250 bp)

pKT25 Two-hybrid plasmid, *cyaAT25* fusion; Karimova *et al*. (1998)

Kan^r^

pUT18 Two-hybrid plasmid, *cyaAT25* fusion; Karimova *et al*. (1998)

Amp^r^

pIJ10434 pKT25 carrying a *Bam*HI/*Asp*718 This work

*bldM* ORF fragment

pIJ10435 pUT18 carrying a *Bam*HI/*Hin*dIII This work

*bldM* ORF fragment

pIJ10436 pKT25 carrying a *Bam*HI/*Asp*718 This work

*whiI* ORF fragment

pIJ10437 pUT18 carrying a *Bam*HI/*Hin*dIII This work

*whiI* ORF fragment

pIJ10444 pIJ10501 carrying *bldM* ORF inframe This work

with the triple FLAG codons

pIJ10445 pMS82 carrying N-terminal triple FLAG This work

*whiI* with native promoter

pIJ10446 pETDuet-1 carrying a *Bam*HI/*Hin*dIII This work

*bldM* ORF fragment and *Nde*I/*Asp*718

*whiI*-ORF fragment

pIJ10447 as pIJ10446 but *whiI* is N-terminally SII- This work

tagged

**References**:

Datsenko KA, Wanner BL (2000) One-step inactivation of chromosomal genes in *Escherichia* *coli* K-12 using PCR products. Proc Natl Acad Sci USA 97: 6640-6645.

Ehrlich J, Gottlieb D, Burkholder PR, Anderson LE, Pridham TG (1948) *Streptomyces* *venezuelae*, N. Sp., the Source of Chloromycetin. *J Bacteriol* 56: 467-477.

Gregory M A, Till R, Smith MC (2003) Integration site for *Streptomyces* phage phiBT1 and development of site-specific integrating vectors. *J Bacteriol* 185: 5320-5323.

Gust B, Challis GL, Fowler K, Kieser T, Chater KF (2003) PCR-targeted *Streptomyces* gene replacement identifies a protein domain needed for biosynthesis of the sesquiterpene soil odor geosmin. Proc Natl Acad Sci USA 100: 1541-1546.

Karimova G, Pidoux J, Ullmann A, Ladant D (1998) A bacterial two-hybrid system based on a reconstituted signal transduction pathway. Proc Natl Acad Sci USA 95: 5752-5756.

Molle V, Buttner MJ (2000) Different alleles of the response regulator gene *bldM* arrest *Streptomyces* *coelicolor* development at distinct stages. Mol microbiol 36: 1265-1278.

MacNeil DJ, Gewain KM, Ruby CL, Dezeny G, Gibbons PH, MacNeil T (1992) Analysis of *Streptomyces* *avermitilis* genes required for avermectin biosynthesis utilizing a novel integration vector. Gene 111: 61-68.

Paget MS, Chamberlin L, Atrih A, Foster SJ, Buttner MJ (1999) Evidence that the extracytoplasmic function sigma factor sigmaE is required for normal cell wall structure in *Streptomyces* *coelicolor* A3(2). J Bacteriol 181: 204-211.

Yanisch-Perron C, Vieira J, Messing J (1985) Improved M13 phage cloning vectors and host strains: nucleotide sequences of the M13mp18 and pUC19 vectors. Gene 33: 103-119.
